# Cholera Infantum

**Published:** 1881-09

**Authors:** 


### SELECTIONS.

### CHOLERA INFANTUM.

BY GALEN, XENIA, OHIO.

This disease commonly affects children from three months to two years, and more, of age, and always in the heated term. Some instances are on record where the disease attacked children beyond the two years; in my own practice I have seen cases of the latter class; notably, those who were allowed to nurse up to this period. And it may be remarked in passing that these cases are always obstinate, and require careful management. Ordinarily the crowded city, with its alleys, inner courts and tenement houses, is the chief theatre of this scourge, but we find it in smaller towns, and even in the country.

The attack is usually ushered in by a diarrhœa, sometimes by vomiting and purging, one or the other, and often both.

In fatal cases, that run their course in a short time, the vomiting is nearly always continuous to the end. When the case is prolonged to two, three and often four, or more weeks, the vomiting subsides, or ceases altogether, and the diarrhœa is left behind. When the attack is very violent, vomiting and purging nearly constant, the stomach rejects all food, even cold water. This stage of the disease is marked by great languor and distress, spasmodic pains in the stomach and bowels, and if relief is not soon obtained, prostration comes on, with cold and clammy skin, pallid, sunken features, half-closed eyes, dilated pupil, insensibility amounting to coma, and death in twenty-four hours to three or four days.

The attack is often attended with febrile symptoms in the protracted cases. In these cases the pulse is small, frequent, weak and corded, mouth hot, tongue furred, the surface of the body heated irregularly, extremities often cold while the trunk and head are heated to a preternatural degree that indicates the gravity of the case beyond a doubt. The pyrexia is generally intermittent, the exacerbations occurring in the evening. It is often accompanied by delirium, and a comatose condition.

The abdomen, though usually sunken or flat, is sometimes swollen and painful on pressure. In the progress of the disease, the patient emaciates rapidly, the flesh becomes flabby and soft, the features shrink, the eyes become sunken in the sockets, the surface is pallid, either cold and clammy, or dry and harsh. In the more advanced stages, various phenomena present themselves. The abdomen is sunken or tumid, the mouth aphthous, the tongue dry and covered by a brownish fur, or red and glossy, petechiæ are observed now and then upon the surface of the body, a vesicular eruption on the thorax, the skin is a dull, dirty, or leaden hue, the conjuntiva appears congested, the weakness extreme, the circulation much enfeebled, great restlessness, rolling from side to side with plaintive cries, coma sets in, and the scene is closed often by convulsions and hydrocephalic symptoms.

From the onset, the child usually sleeps, with the eyes more or less open; has thirst, fluids being swallowed with avidity; the appetite is capricious, sometimes entirely wanting, sometimes ravenous, often demanding unnatural articles of food.

The discharges are very various. At first the contents of the stomach and bowels, the matter vomited, is modified by the food frequently mingled with milk, which is generally coagulated.

After the discharge of the feculent matter, the dejections are usually watery and copious, tinged with green, yellow or brown, and often are emerald green. Now and then may be observed hard or concrete matter, or semi-concrete matter, yellow, green, white, or gelatinous, and often tinged with blood. Again, slime, or mucus, comprises the greater part of the evacuations.

Farther in the progress of the disease, we have dark red dejections, resembling washings of flesh. The odor of the discharges is generally offensive, sour, putrid, and very rarely the healthy fecal smell.

When the disease has assumed the form of a lingering diarrhœa, some appetite is present, but very little digestion, the ingesta passing but little changed.

Dr. Dewees says the passing of live worms is unfavorable.

If the patient is attacked in the latter part of the summer, and the strength is not too much enfeebled, the temperature of the air lowered, and no return of relapse, the case may convalesce. The struggle is often one of time, the aim being to keep the enemy at bay till permanent cool weather. If the brain complications can be warded off, the prognosis is favorable, especially if the discharges return to a healthy condition. A regular appetite, diminution of the frequency of the evacuations, a general brightening of the countenance, and cessation of restlessness, are favorable prognostic signs.

Dr. Condie says if death occurs early, the mucous coat. and more or less hepatic congestion, are the morbid appearances.

In the *American Journal of Medical Science*, a writer observes that "there is undue development of the follices, both of the stomach and intestines."

At a more advanced stage of the disease there is an indication of inflammation of the mucous membrane of the stomach and bowels, red patches, and ulceration both in large and small intestines. Livid spots are often observed by pathologists upon the stomach and duodenum. Dr. Horner saw pus in the colon in one case, and retained fecal matter in the large intestines. The liver is generally enlarged and more or less congested. The gall-bladder contains a liquid which is sometimes dark-green, and sometimes pale color.

The brain is often found in a congested state, with serous effusion in the ventricles, and thickening of the arachnoid. Dr. Lindsey found the bladder empty and contracted.

Dr. J. Lewis Smith found stomach healthy in some, while the intestines showed inflammation, the color being uniformly reddened and thickened; others exhibited follicular ulceration.

*Ohio State Journal of Dental Science.*

